# Transporting Patient with Suspected SARS

**DOI:** 10.3201/1007.030608

**Published:** 2004-07

**Authors:** Shin-Han Tsai, Chiu-Man Tsang, Hsueh-Ru Wu, Li-Hua Lu, Yung-Chia Pai, Mark Olsen, Wen-Ta Chiu

**Affiliations:** * National Aeromedical Consultation Center, Taipei, Taiwan;; † Taipei Medical University, Taipei, Taiwan;; ‡ Wan Fang Hospital, Taipei, Taiwan;; § International SOS, Taipei, Taiwan;; ¶ Chun Shan Hospital, Taipei, Taiwan;; # Mackay Memorial Hospital, Taipei, Taiwan

**Keywords:** infection control, infectious diseases, emerging, communicable disease, Severe acute respiratory syndrome, aviation medicine, Air ambulance, Isolation, Patient

**To the Editor:** The severe acute respiratory syndrome (SARS) outbreak in Taiwan can be traced back to a Taiwanese businessman who returned from mainland China to Taiwan in March 2003 (1). In May 2003, several outer islands belonging to Taiwan reported SARS, and on June 2, 2003, Penghu Army Hospital reported a 40-year-old man with suspected SARS. The patient complained of shortness of breath and a dry cough. He had visited a person with confirmed SARS 7 days earlier. He had a temperature of 38.4°C and leukocyte count of 7,920 cells/µL, and his chest x-ray showed infiltration in both lower lobes.

Because medical facilities are limited on these islands, the Department of Health authorized the National Aeromedical Consultation Center (NACC), a physician-based 24-hour control center that coordinates all aeromedical transport of critically ill or injured patients within Taiwan, to coordinate transporting these patients to designated SARS hospitals in Taipei. The NACC dispatched an aircraft (Fokker 50) with a specialized team of two flight physicians, one flight paramedic, and a PIU (portable isolation unit) on board. During the flight, the medical crew prepared equipment and dressed themselves in three layers of personal protective equipment. On arrival at Penghu, only essential equipment was taken into the hospital. One physician took the PIU into the isolation room. The rest of the crew and equipment remained in the pre-isolation room. The patient was briefed about the transport and given 10 mg of metoclopramide to prevent motion sickness. He was asked to get into the PIU. A pulse oximeter was attached to his finger and placed inside the PIU so that it could be read from the outside. A thermohydrometer was also placed inside the unit. The patient was given a squeeze-bottle of water, and the unit was sealed and inflated.

When leaving the pre-isolation room, the physician and the PIU were sprayed with a sodium hypochloride solution before the first layer of personal protective equipment was removed. At the exit, the entire medical crew removed a layer of personal protective equipment after being sprayed with sodium hypochloride solution. The team returned to the airport for the flight back to Taiwan. No other personnel or family member was allowed to accompany the patient on the flight.

The patient remained stable and calm throughout the flight. His oxygen saturation remained 97%–99% with heart rate of 90 to 100 beats per minute. Humidity was maintained at 60% and temperature at 28°C. On arrival, the team proceeded to the isolation ward. The physician accompanied the patient into the isolation room; the patient was released from the PIU and transferred to the receiving medical team.

On exiting the isolation room, the empty PIU and the medical team were sprayed with sodium hypochloride. All equipment was sprayed and put into biohazard bags. The medical team then discarded the last layer of impermeable clothing. The PIU was left in biohazard bags for 24 hours before being sprayed with water and air-dried.

After the assignment, the medical crew self-documented their temperature twice daily for 10 days. All staff remained asymptomatic with normal body temperatures during this period. The patient’s temperature remained normal, and results of a polymerase chain reaction of throat swab were negative for SARS-associated coronavirus (SARS-CoV). He was discharged on June 10, 2003.

When the SARS outbreak occurred in Taiwan, many medical and ambulance personnel were exposed to SARS-CoV while transporting or caring for patients with suspected SARS. As SARS was an emerging infectious disease, the mechanism of transmission was still unclear. Although one report by Christopher and Eitzen (2) suggested the value of an aeromedical team to evacuate patients with suspected lethal, infectious diseases, limited evidence supported a safer means of transportation that would possibly reduce transmission of SARS to persons taking part in the mission.

When the SARS epidemic spread to remote islands, aircraft companies refused to transport patients with a case of suspected SARS unless certain precautions were implemented. Smaller aircraft used on domestic routes in Taiwan do not meet the standards set for transporting SARS patients (3,4), which prompted the design of the PIU, an airtight polyvinyl chloride bag with a one-way inlet valve and an exhalation valve. The valves were modified by incorporating HEPA filters on both sides of the valves and then connecting a ventilator with an oxygen source to the inlet valve. The respiratory rate and tidal volume are set, depending on weight and oxygen requirements of the patient. By regulating the exhalation valve, the minimum pressure inside the bag can be manipulated to keep it from collapsing, since the bag has no internal or external frame.

The PIU has some limitations. No physical contact with the patient is possible after the PIU is sealed and inflated. Very strict criteria on the suitability of a patient to be transported are followed. Any patient who is unconscious, uncooperative, or whose condition may deteriorate is not transportable in this unit. Because of possible discomfort, a maximum total transport time of 2 to 4 hours is suggested. This time frame works well in Taiwan; all locations in the country, including the outer islands, are within a 4-hour limit.

The use of PIU during the SARS crisis had a number of positive effects in Taiwan. It enabled the safe transport of SARS patients between hospitals by air and road and decreased the risk of cross-infecting transport personnel. The anxiety of transport personnel was decreased, as was the fear felt by the population of the outer islands. In addition, the credibility of the local health authorities was improved among the general population in Taiwan.
